# History of Alcohol and Opioid Use Impacts on the Long-Term Recovery Trajectories of Methamphetamine-Dependent Patients

**DOI:** 10.3389/fpsyt.2019.00398

**Published:** 2019-06-07

**Authors:** Haoye Tan, Di Liang, Na Zhong, Yan Zhao, Zhikang Chen, Min Zhao, Haifeng Jiang

**Affiliations:** ^1^Shanghai Mental Health Center, Shanghai Jiao Tong University School of Medicine, Shanghai, China; ^2^Department of Family Medicine and Public Health, University of California, San Diego, CA, United States; ^3^Shanghai Key Laboratory of Psychotic Disorders, Shanghai, China

**Keywords:** long-term follow-up, recovery pattern, negative consequences, trajectory, methamphetamine

## Abstract

Methamphetamine (MA) has become one of the most widely used illicit substances in China and the rest of the world as well. Relapse, incarceration or death was observed after compulsory rehabilitation. However, the knowledge of recovery patterns among MA-dependent patients, early or late occurrence of these negative consequences, is limited. The aims were to explore the long-term recovery patterns and associated factors among MA-dependent patients in Shanghai, China. MA-dependent patients discharged from Shanghai compulsory rehabilitation facilities in 2009–2012 were recruited in a baseline survey. The baseline data of 232 patients were then linked with their long-term follow-up data from official records. Group-based trajectory modeling was applied to identify distinctive trajectories of the occurrence of negative consequences (incarceration, or readmission to compulsory rehabilitation, or death). Patients with monthly status data were found recovering with three distinctive trajectories: rare, late, and early occurrence groups. Multinomial logistic regression showed that having alcohol use history was associated with an increased likelihood of being in the late occurrence group relative to the rare occurrence group. Having opioid use history was associated with an increased likelihood of being in the early occurrence group relative to the rare occurrence group. In addition, being female was associated with decreased likelihood of being in the late occurrence group relative to the rare occurrence group.

## Introduction

While ranking second in the share of the global burden of disease attributable to drug use disorders after opioids, amphetamine-type stimulants (ATS) are the most frequently used class of illicit drugs in China, and people using opioids also gradually switched to ATS (1, 2). Methamphetamine (MA) is the primary drug used among ATS. MA dependence has a relapse rate of 30%–90%, and a study in 2014 showed that 61% of the MA users relapsed within 1 year following treatment discharge (3–5). MA use is also linked to crime, such as drug dealing, property crime, fraud or violent crime, especially acquisitive crime (6–9).

Relapse is associated with more than a single factor. Previous studies found that both biological and sociocultural characteristics of patients could influence relapse (10, 11). It was revealed that some demographic factors such as age, gender and education level related to relapse among ATS users (12, 13). Meanwhile, patients’ mental comorbidities, having psychotic symptoms and polydrug use, were risk factors or protective factors (13–15). Crime is also related to a combination of drug use and sociocultural characteristics (12). It was found that frequent drug and alcohol use were risk factors for incarceration among Thai MA users (16). Furthermore, there was a picture of mutual influence between relapse and crime. Among Japanese patients with MA use disorder, history of incarceration was associated with treatment retention (13).

In China, there is a compulsory treatment program, according to the Chinese narcotic control law, for patients who fail to remain abstinent from drug use in the community. The compulsory rehabilitation program is an enforced residential drug treatment. Thus, participants in this study maintained abstinence from entrance to discharge. It is conducted by a judicial office, and the patient who has an addiction to illicit drugs may be sent to obtain a compulsory treatment, which is usually for 2 years. This compulsory treatment program aimed for comprehensive recovery of physical health (daily physical exercise), from drug dependence and of social functioning, which includes medication or physical rehabilitation, psychotherapy and vocational skills training and anti-relapse education. In this program, they received no medications related to drug dependence. When the compulsory treatment program is completed, there are social worker networks to prevent relapse and crime and promote social functioning recovery (17). Patients who are discharged from the compulsory treatment program are assigned to participate in the community-based drug rehabilitation program that serves at their place of residence. After the compulsory treatment program, patients will participate in the community-based drug rehabilitation program, and social workers could provide psychological counseling, vocational training and social welfare consultation, which is funded by the government (18). Therefore, to assess the comprehensive recovery of patients after the treatment using the Chinese model, we define negative consequences (NC) (including incarceration, readmission to compulsory rehabilitation and death) to assess rehabilitation, which was used in our previous study among heroin patients (19).

In community-based rehabilitation, patients have different recovery trajectories. Some patients have NC, while others abstinence. The time points of NC occurrence were different, which range from a few months to years in our observation. However, a few research has indicated how the trajectory develops and what factors affect rehabilitation trajectories. Recently, there was a nationwide systematic multicenter survey of the characteristics of drug use behaviors in club drug users and associated high-risk sexual behavior in China, which showed that the pursuit of euphoria was the main reason for drug use. High-risk sexual behaviors were common in these users. The factors of polydrug use, long use history and severe acute intoxication after drug use were associated with risky sexual behaviors. With this survey, this study has required part of the baseline data to explore the recovery trajectories (20). We investigated recovery patterns of MA-dependent patients and associated risk factors, based on an electronic monthly summary record system of persons using illicit drugs in Shanghai, China. This follow-up database, which was established by Shanghai Municipal Narcotics Control Committee, provided us a unique opportunity to describe the recovery patterns among MA-dependent patients. We used group-based trajectory modeling (GBTM) to identify distinctive trajectories of the presence of NC after patients were discharged from compulsory rehabilitation programs.

## Methods

### Design

This study was a cohort study. The baseline data were collected from the project “Research on mathematical model for AIDS epidemic trend assessment and prediction in China” (20). After the baseline assessment, our participants were passively followed up: participants’ long-term outcomes were ascertained from the electronic monthly summary record system, which was managed by social workers. Unique ID numbers were used to link baseline data to follow-up data.

This study was approved by the institutional Review Boards in Shanghai Mental Health Center. Written informed consent was obtained from all participants. All procedures were in accordance with the approved guidelines.

### Participants

**Figure 1:**
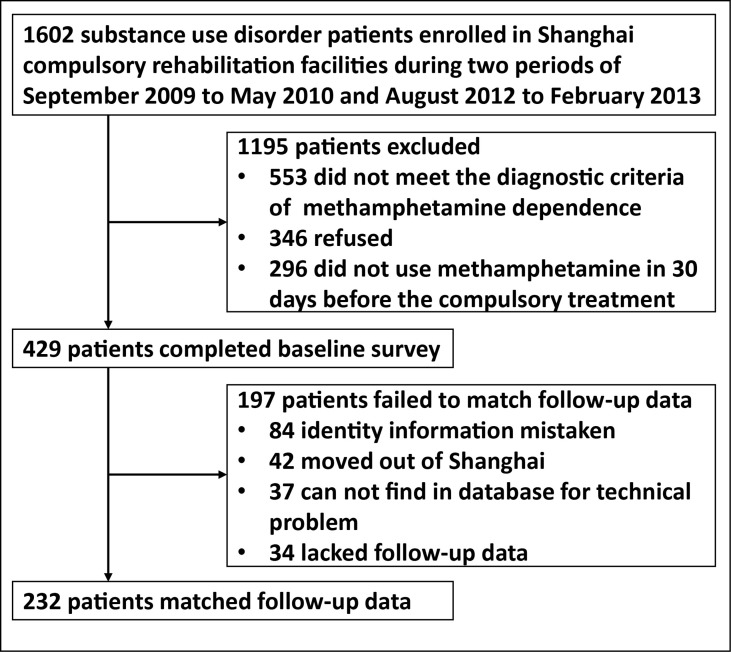
Flowchart of enrollment and follow-up of subjects in the study.

At baseline, we used convenience sampling to recruit 429 MA-dependent patients from two compulsory rehabilitation centers in Shanghai from September 2009 to May 2010 and from August 2012 to February 2013. Patients who met the inclusion criteria should: a) have MA dependence according to the *Diagnostic and Statistical Manual of Mental Disorders, 4th Edition* diagnostic criteria; b) be at the age of 18 and above; c) have used MA in 30 days (by urinalysis) before the mandatory drug rehabilitation; and d) have the ability of informed consent. The exclusion criteria were: a) serious physical or neurological illness that required pharmacological treatment; b) other Axis I disorder of the *Diagnostic and Statistical Manual of Mental Disorders, 4th Edition* criteria, such as bipolar disorder, schizophrenia, depression and substance dependence (other than nicotine and MA) within the past 5 years; and c) neurological diseases, such as stroke, seizure, migraine, and head trauma. The process is displayed in **Figure 1**.

### Measurements

At baseline, a commonly used Chinese version of the Addiction Severity Index (ASI) was used to assess patients’ sociodemographic characteristics and drug use history, including age, sex, employment, marriage, alcohol/drug usage and so on (21, 22).

The follow-up data of participants after compulsory rehabilitation programs were derived from the electronic monthly summary record system from 2009 to 2017. Shanghai Municipal Narcotics Control Committee established this database in March 2007. Social workers who are employed by the Shanghai government are responsible for managing this administrative database, while helping the patients with drug dependence recover in the community. The following categories of drug-related information were recorded each month in chronological order, including incarceration, readmission to compulsory rehabilitation programs, death, methadone maintenance treatment participation, etc. These events were recorded as binary variables (happened or not).

For the present study, recovery outcomes were classified into four monthly outcomes: incarceration, readmission to compulsory rehabilitation programs, death, and the rest (not encoded as any of other cases). Our outcome variable of interest, negative consequences, is defined as having incarceration, readmission to compulsory rehabilitation programs or death. According to the Anti-drug Law in China (17), patients who were discharged from compulsory rehabilitation programs should participate in a long-term community-based rehabilitation program. During the community-based rehabilitation program, using illicit drugs could lead to readmission into compulsory rehabilitation programs. Readmission was only triggered by seriously violating the community-based recovery agreement or reusing drugs, which means those readmission cases relapsed. According to clinical observation, we hypothesized the recovery patterns of patients, which can be divided to the following: 1) NC happened in a relatively short time after compulsory rehabilitation programs (early occurrence group); 2) NC happened long after compulsory rehabilitation programs (late occurrence group); and 3) NC rarely happened (rare occurrence group).

### Statistical Analyses

Group-based trajectory modeling (GBTM) was used to analyze the patient’s recovery trajectories. GBTM is a specialized application of finite mixture modeling. In this study, GBTM identifies clusters of individuals with similar recovery trajectory and explores heterogeneity across groups. The monthly repeated measures of NC were estimated by a polynomial relationship as below:

$${\text{Status}}_{ijt}=\beta_{0j}+\beta_{ij}\times Month_{it}+\beta_{2j}\times Month_{it2}+\varepsilon_{it}$$

Where *i*,* j*, and *t* indicate subjects, latent group, and time, respectively, and ε is a disturbance normally distributed with a zero mean and a constant residual variance.

The shape of trajectory for each group determined β_0_, β_1_ and β_2_, which represent the intercept, linear and quadratic parameters, respectively. (23, 24). We used ‘traj’ plugin in Stata release 12 for analysis (25–27). Corresponding to the binary variables of the recovery data, the Logistic Model was used. A series of models were fitted with an increasing number of trajectory groups. The goodness of fit model was evaluated with the Bayesian Information Criterion (BIC) (28). The best fitting model was chosen with a reasonably low absolute value of BIC and sufficient number of subjects (10% of total sample or more) in each group (29).

For the subgroups with separated trajectories, multinomial logistic regression was used to explore the relationship between one’s recovery pattern and baseline factors.

## Results

### Attrition and Characteristics of Patients

Among 429 participants, 232 patients’ follow-up data were found in the official database and were linked to their baseline data. The rest of the participants’ follow-up data were not found due to mistaken identity, those who moved out of Shanghai or technical problems (see **Figure 1**). Among all 429 patients, 68.2% were male; 46.9% were unemployed or had spent time in prison in the 3 years prior to the baseline interview; over half (60.3%) were not currently married; they had, on average, a 3.1 ± 2.6-year history of MA use, and in 30 days before compulsory rehabilitation, they had 15.0 ± 12.0 times of drug use on average (see **Supplementary Table 1**).

### Status During 30 Months of Follow-Up and Recovery Patterns

**Figure 2:**
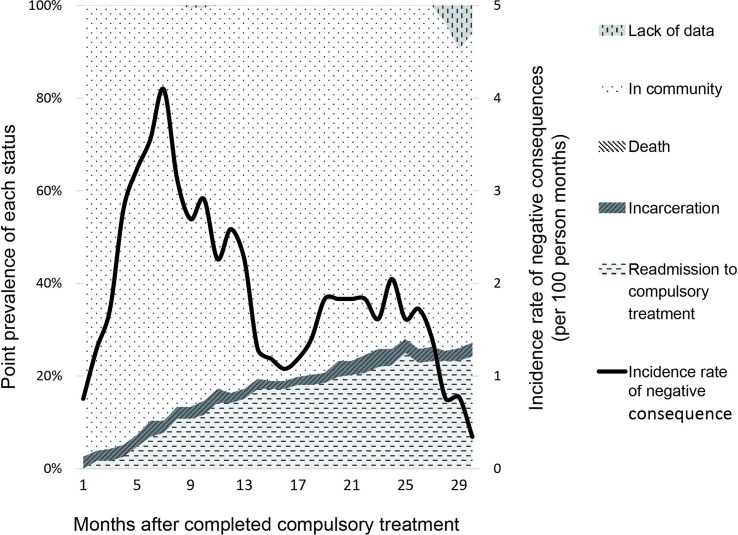
Recovery status of methamphetamine patients after completed the compulsory rehabilitation program in Shanghai, China.The line displayed the incidence rate (per 100 person months) and the area in the figure showed the monthly prevalence of each negative consequence (incarceration, readmission to compulsory treatment and death). There was no death case that happened during this period.

As participants were discharged at different time, our follow-up data ranged from 30 to 86 months. Thus, we truncated the first 30 months after compulsory rehabilitation programs for analysis. The monthly prevalence of each NC and incidence rates of all NC after compulsory rehabilitation are graphically displayed in **Figure 2**. Incidence rates were calculated by person-time methods, dividing the number of NC by the number of NC and in community monthly. Two peaks of NC were observed. Thus, we confirmed our hypothesis that the recovery patterns of patients can be divided as early occurrence group, late occurrence group and rare occurrence group.

**Figure 3:**
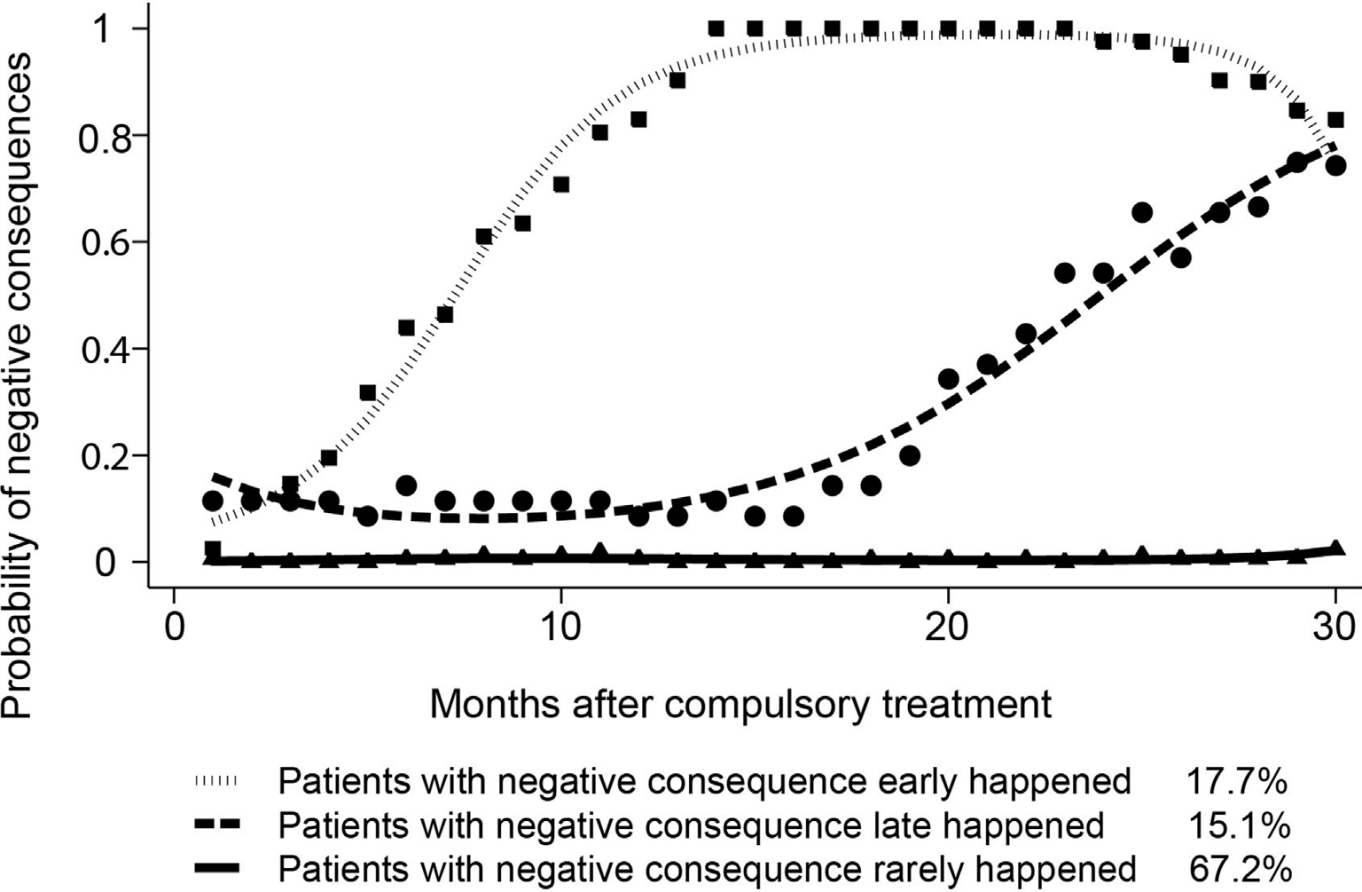
Trajectories as defined by group-based trajectory modeling (GBTM) analysis of recovery status over time.Three-trajectory group-based trajectory modeling was used to fit the recovery data of methamphetamine patients and predict possibility of negative consequences. Negative consequences included incarceration, readmission to compulsory treatment and death.

A series of group-based trajectory models, from a two- to five-trajectory pattern, were fitted to identify the optimal model. The BIC values (BIC = −1,538.40) in the two-trajectory model, three (BIC =−1,236.76), four (BIC = −1,145.07) and five (BIC = −1,046.75) were used for model evaluation. To evaluate class separation, the relative entropy of the posterior probability distribution was calculated and had a value of 0.8, indicating the acceptable separation between classes (30). When the relatively low absolute value of BIC and the sufficient number of subjects in each group and clinical interpretability were considered, the three-trajectory model was selected as potential optimal models. The trajectories and baseline characteristics are displayed in **Figure 3** and **Table 1**, respectively.

**Table 1:** The social demographic and drug use characteristics of the participants with follow-up.

	Total *n* = 232, (100%)	Rare occurrence group *n* = 156, (67.2%)	Late occurrence group *n* = 35, (15.1%)	Early occurrence group *N* = 41, (17.7%)
Demographic characteristics				
Age, years (mean, std)	35.62, (8.24)	36.21, (8.55)	34.66, (6.71)	34.24, (8.19)
Gender				
Male (*n*, %)	146/227, (64.3%)	92/152, (60.5%)	28/35, (80%)	26/40, (65%)
Female (*n*, %)	81/227, (35.7%)	60/152, (39.5%)	7/35, (20%)	14/40, (35%)
Ethnicity				
Han (*n*, %)	225/231, (97.4%)	150/155, (96.8%)	35/35, (100%)	40/41, (97.6%)
Others (*n*, %)	6/231, (2.6%)	5/155, (3.2%)	0/35, (0%)	1/41, (2.4%)
Employment				
Employed (*n*, %)	117/230, (50.9%)	81/156, (51.9%)	18/34, (52.9%)	18/40, (45%)
Unemployed (*n*, %)	113/230, (49.1%)	75/156, (48.1%)	16/34, (47.1%)	22/40, (55%)
Currently married				
Yes (*n*, %)	88/229, (38.4%)	62/154, (40.3%)	13/34, (38.2%)	13/41, (31.7%)
No (*n*, %)	141/229, (61.6%)	92/154, (59.7%)	21/34, (61.8%)	28/41, (68.3%)
Accommodation				
Live with parents or children (*n*, %)	98/229, (42.8%)	67/153, (43.8%)	18/35, (51.4%)	13/41, (31.7%)
Live alone or with others (*n*, %)	131/229, (57.2%)	86/153, (56.2%)	17/35, (48.6%)	28/41, (68.3%)
Education				
Less than high school (*n*, %)	152/229, (66.4%)	107/153, (69.9%)	19/35, (54.3%)	26/41, (63.4%)
High school (*n*, %)	65/229, (28.4%)	38/153, (24.8%)	15/35, (42.9%)	12/41, (29.3%)
More than high school (*n*, %)	12/229, (5.2%)	8/153, (5.2%)	1/35, (2.9%)	3/41, (7.3%)
Education experience, years (mean, std)	9.48, (2.14)	9.39, (2.08)	9.80, (2.06)	9.51, (2.41)
Drug use history				
Use history, years (mean, std)	2.91, (2.62)	2.85, (2.73)	3.00, (2.54)	3.05, (2.28)
Onset age, years (mean, std)	32.75, (8.75)	33.42, (9.09)	31.66, (7.77)	31.2, (8.15)
30 days frequency, times (mean, std)	13.85, (12.04)	13.31, (11.89)	15.45, (12.09)	14.4, (12.7)
Opioid use history				
Yes (*n*, %)	89/203, (43.8%)	53/133, (39.8%)	15/34, (44.1%)	21/36, (58.3%)
No (*n*, %)	114/203, (56.2%)	80/133, (60.2%)	19/34, (55.9%)	15/36, (41.7%)
Marijuana use history				
Yes (*n*, %)	55/232, (23.7%)	34/156, (21.8%)	9/35, (25.7%)	12/41, (29.3%)
No (*n*, %)	177/232, (76.3%)	122/156, (78.2%)	26/35, (74.3%)	29/41, (70.7%)
Use with partner				
Yes (*n*, %)	45/229, (19.7%)	33/153, (21.6%)	5/35, (14.3%)	7/41, (17.1%)
No (*n*, %)	184/229, (80.3%)	120/153, (78.4%)	30/35, (85.7%)	34/41, (82.9%)
Alcohol use^a^				
Yes (*n*, %)	74/203, (36.5%)	42/133, (31.6%)	16/34, (47.1%)	16/36, (44.4%)
No (*n*, %)	129/203, (63.5%)	91/133, (68.4%)	18/34, (52.9%)	20/36, (55.6%)

a Regularly drink (more than 15 days per month) more than 1 year before compulsory rehabilitation.

### Associated Factors of Negative Consequences

When distinct trajectories were identified by GBTM, multinomial logistic regression was used to explore the relationship between one’s recovery pattern and baseline characteristics. Regression results are presented in **Table 2**. Having alcohol use history (use more than 15 days per month) was associated with the increased likelihood (OR = 2.74, *p* = 0.027) of being in the late occurrence group relative to the rare occurrence group. Having opioid use history was associated with increased likelihood (OR = 2.35, *p* = 0.053) of being in the early occurrence group relative to the rare occurrence group, although the estimation association was marginally significant. In addition, being female was associated with the decreased likelihood (OR = 0.37, *p* = 0.051) of being in the late occurrence group relative to the rare occurrence group, and the association was also marginally significant.

**Table 2:** Multinomial logistic regression analysis of the three recovery trajectory groups.

	Odds ratio of late occurrence vs. rarely (95% confidence interval)	Odds ratio of early occurrence vs. rarely (95% confidence interval)
Demographic characteristics		
Females (vs. Males)	0.35 (0.12,1.00)	0.72 (0.30,1.74)
Employed (vs. Unemployed)	1.40 (0.58,3.38)	0.81 (0.35,1.86)
Current married (vs. Current unmarried)	0.55 (0.22,1.38)	0.65 (0.27,1.55)
Accommodation^a^	1.48 (0.62,3.53)	0.89 (0.38,2.10)
Drug use history		
Use daily (vs. lower frequency)	0.77 (0.29,2.03)	0.89 (0.36,2.22)
Use year (vs. < 1 year)		
1 year	0.54 (0.13,2.32)	0.28 (0.06,1.26)
2–5 year	0.70 (0.20,2.52)	0.72 (0.23,2.32)
>5 years	1.91 (0.39,9.42)	0.98 (0.20,4.72)
Opioid use history (vs. not)	1.41 (0.59,3.37)	2.31 (0.99,5.40)
Marijuana use history (vs. not)	1.09 (0.38,3.08)	1.46 (0.56,3.81)
Alcohol use^b^	2.74 (1.12,6.73)*	2.30 (0.94,5.64)

a Live with parents or children vs. live alone or with others.

b Regularly drink more than 1 year vs. occasionally or never drink.

*p < 0.05.

## Discussion

To our knowledge, this is the first study examining recovery patterns among MA-dependent patients after compulsory rehabilitation programs in China. Our findings extended our knowledge of long-term recovery trajectories of MA-dependent patients and associated factors. By using GBTM, we identified three groups among MA-dependent patients: early occurrence group, late occurrence group, and rare occurrence group. Alcohol use history, opioid use history and being female might be associated with patients’ recovery trajectories.

We found that baseline alcohol use history was associated with increased likelihood of being in the late occurrence group relative to the rare occurrence group, and baseline opioid use history was associated with increased likelihood of being in the early occurrence group relative to the rare occurrence group. These findings were consistent with previous studies showing that drug use disorders were closely associated with alcohol use (31). In addition, heavy alcohol consumption increased the risk of violent behaviors, and alcohol use accounted for 12–18% of the violence risk related to MA use (32). Violent behaviors might result in incarceration, which is another aspect of NC. Moreover, brain image research of functional links in valuation networks demonstrated that heroin abstinence could influence functional connectivity and resulted in impulsive behaviors (33). A recent animal study showed that the sensitivity to opioids, which involved the mu-opioid receptor (MOP-r) regulated systems, has a negative genetic correlation with MA consumption in mice (34). This indicated that opioid sensitivity and MA intake were genetically associated, and opioid-mediated pathways influence MA use. Previous studies found that in long-term opiate abusers, the function of the MOP-r is altered in response to its ligands (35–37). It is also observed that MA use was a problem in patients in methadone maintenance treatment (38, 39). Furthermore, in patients with severe alcoholism, a neuroadaptation to an alcohol-induced release of endogenous ligands appeared to reduce MOP-r (40). The change of MOP-r might be related to the higher risk of relapse, the main part of NC. It would be interesting to explore in future research whether what we found in the current study that alcohol and opioid use history predicted the occurrence of NC was related to the severity of MOP-r dysfunction. Our findings also suggested that being female was associated with decreased likelihood of being in the late occurrence group relative to the rare occurrence group. This was consistent with our previous study with heroin-dependent patients, which was found that female patients were less likely to experience negative outcomes than male patients (19).

In our results, when opioid and alcohol use affected the occurrence of NC, social and family factors did not seem to have a critical impact on it. This suggests that neurobiological changes caused by poly-substance use may have a greater impact on long-term rehabilitation. Therefore, the experience of pharmacotherapy in alcohol and opioid dependence could enlighten long-term NC prevention in MA dependence (41, 42), and it might be necessary to address the problem of poly-substance use that shares the similar neurobiological change.

Our study had several limitations. First, although NC might be better than considering relapse or crime separately in assessing recovery trajectory, readmission in NC could cause underestimation of relapse because we could not directly measure patients’ relapse. Thus, our outcome of readmission might not be an ideal proxy for relapse, as patients may relapse but were not found by social workers or readmitted to a compulsory rehabilitation center. In addition, we did not have data on patients’ mental health, healthcare obtained, and other characteristics that changed with time, which might also impact on patients’ recovery trajectory. Furthermore, our results might not be generalized to MA-dependent patients in other areas of China, considering regional variations across China, or those who were not admitted to compulsory rehabilitation programs.

In conclusion, MA-dependent patients presented various recovery patterns after being discharged from compulsory rehabilitation programs in Shanghai, China. When caring for MA-dependent patients, healthcare providers should take patients’ alcohol use problem into consideration to prevent the occurrence of NC. Future prevention and early intervention of NC should also consider more about patients with the history of poly-substance use.

## Data Availability Statement

The datasets for this study will not be made publicly available because the authors do not have permission to share data.

## Ethics Statement

This study was carried out in accordance with the recommendations of name of guidelines, name of committee with written informed consent from all subjects. All subjects gave written informed consent in accordance with the Declaration of Helsinki. The protocol was approved by the name of committee.

## Author Contributions

MZ and HJ designed this study, and all the authors participated in this process. NZ, YZ and ZC collected the data, and HT and DL analyzed the data and drafted the manuscript. All the authors edited the paper.

## Funding

The authors thank the sponsors of National Key R&D Program of China [2017YFC1310400], Natural Science Foundation of China [81601164, U1502228, 81771436], Clinical Research Center, Shanghai Jiao Tong University School of Medicine [DLY201818], Science and Technology Commission of Shanghai Municipality [18411961200], Municipal Human Resources Development Program for Outstanding Young Talents in Medical and Health Sciences in Shanghai [2017YQ013], Shanghai Mental Health Center Flyer Plan [2016FX003], Program of Shanghai Academic Research Leader [17XD1403300], Shanghai Key Laboratory of Psychotic Disorders [13dz2260500] and Shanghai Municipal Health and Family Planning Commission (2017ZZ02021).

## Conflict of Interests Statement

The authors declare that the research was conducted in the absence of any commercial or financial relationships that could be construed as a potential conflict of interest.
