# Supplementary material for: History of Alcohol and Opioid Use Impacts on the Long-Term Recovery Trajectories of Methamphetamine-Dependent Patients
Source: Front Psychiatry. 2019 Jun 7;10:398. doi: 10.3389/fpsyt.2019.00398 (PMC6568272; doi:10.3389/fpsyt.2019.00398)
Supplement: Supplementary file 1 [file Table_1.docx]

Supplementary Table 1. The social demographic and drug-use characteristics of the all participants.

|  | Total  n= 429, (100%) | follow-up data matched  n= 232, (54.1%) | follow-up data unmatched  n= 197, (45.9%) |
| --- | --- | --- | --- |
| Demographic characteristics | | | |
| Age, years (Mean, std) | 35.29, (8.32) | 35.62, (8.24) | 34.91, (8.41) |
| Gender |  |  |  |
| Male (n, %) | 288/422, (68.2%) | 146/227, (64.3%) | 142/195, (72.8%) |
| Female (n, %) | 134/422, (31.8%) | 81/227, (35.7%) | 53/195, (27.2%) |
| Ethnicity |  |  |  |
| Han (n, %) | 417/428, (97.4%) | 225/231, (97.4%) | 192/197, (97.5%) |
| Others (n, %) | 11/428, (2.6%) | 6/231, (2.6%) | 5/197, (2.5%) |
| Employment |  |  |  |
| Employed (n, %) | 225/424, (53.1%) | 117/230, (50.9%) | 108/194, (55.7%) |
| Unemployed (n, %) | 199/424, (46.9%) | 113/230, (49.1%) | 86/194, (44.3%) |
| Currently married |  |  |  |
| Yes (n, %) | 169/426, (39.7%) | 88/229, (38.4%) | 81/197, (41.1%) |
| No (n, %) | 257/426, (60.3%) | 141/229, (61.6%) | 116/197, (58.9%) |
| Accommodation |  |  |  |
| Live with parents or children (n, %) | 156/425, (36.7%) | 98/229, (42.8%) | 58/196, (29.6%) |
| Live alone or with others (n, %) | 269/425, (63.3%) | 131/229, (57.2%) | 138/196, (70.4%) |
| Education |  |  |  |
| Less than high school (n, %) | 275/426, (64.6%) | 152/229, (66.4%) | 123/197, (62.4%) |
| High school (n, %) | 121/426, (28.4%) | 65/229, (28.4%) | 56/197, (28.4%) |
| More than high school (n, %) | 30/426, (7%) | 12/229, (5.2%) | 18/197, (9.1%) |
| Education experience, years (Mean, std) | 9.44, (2.38) | 9.48, (2.14) | 9.4, (2.64) |
| Drug use history | | | |
| Use history, years (Mean, std) | 3.14, (2.64) | 2.91, (2.62) | 3.41, (2.65) |
| Onset age, years (Mean, std) | 32.11, (8.68) | 32.75, (8.75) | 31.34, (8.55) |
| 30 Days frequency, times (Mean, std) | 15.01, (11.98) | 13.85, (12.04) | 16.35, (11.79) |
| Opioid use history |  |  |  |
| Yes (n, %) | 178/388, (45.9%) | 89/203, (43.8%) | 89/185, (48.1%) |
| No (n, %) | 210/388, (54.1%) | 114/203, (56.2%) | 96/185, (51.9%) |
| Marijuana use history |  |  |  |
| Yes (n, %) | 98/429, (22.8%) | 55/232, (23.7%) | 43/197, (21.8%) |
| No (n, %) | 331/429, (77.2%) | 177/232, (76.3%) | 154/197, (78.2%) |
| Use with partner |  |  |  |
| Yes (n, %) | 88/421, (20.9%) | 45/229, (19.7%) | 43/192, (22.4%) |
| No (n, %) | 333/421, (79.1%) | 184/229, (80.3%) | 149/192, (77.6%) |
| Alcohol use |  |  |  |
| Yes (n, %) | 130/388, (33.5%) | 74/203, (36.5%) | 56/185, (30.3%) |
| No (n, %) | 258/388, (66.5%) | 129/203, (63.5%) | 129/185, (69.7%) |
| 1. Regularly drink more than 1 year before compulsory rehabilitation | | | |
